# Modification of Polyethersulfone Ultrafiltration Membrane Using Poly(terephthalic acid-co-glycerol-g-maleic anhydride) as Novel Pore Former

**DOI:** 10.3390/polym14163408

**Published:** 2022-08-20

**Authors:** Ali A. Abbas Aljanabi, Noor Edin Mousa, Mustafa M. Aljumaily, Hasan Sh. Majdi, Ali Amer Yahya, Mohammad N. AL-Baiati, Noor Hashim, Khaild T. Rashid, Saad Al-Saadi, Qusay F. Alsalhy

**Affiliations:** 1 Al-Mussaib Technical College, Al-Furat Al-Awsat Technical University, Babylon 51009, Iraq; 2 Membrane Technology Research Unit, Chemical Engineering Department, University of Technology-Iraq, Alsena’a Street No. 52, B. O. 35010, Baghdad 10066, Iraq; 3 Department of Civil Engineering, Al-Maarif University College, Al-Ramadi 31001, Iraq; 4 Department of Chemical Engineering and Petroleum Industries, Al-Mustaqbal University College, Babylon 51001, Iraq; 5 Department of Chemistry, College of Education for Pure Sciences, University of Kerbala, Holly Kerbala 56001, Iraq; 6 Department of Mechanical and Aerospace Engineering, Monash University, Clayton, VIC 3800, Australia

**Keywords:** polyethersulfone, poly(terephthalic acid-co-glycerol-g-maleic anhydride), ultrafiltration membrane, water-soluble, pore former, bovine serum albumin, sodium alginate

## Abstract

In this research, poly terephthalic acid-co-glycerol-g-maleic anhydride (PTGM) graft co-polymer was used as novel water-soluble pore formers for polyethersulfone (PES) membrane modification. The modified PES membranes were characterized to monitor the effect of PTGM content on their pure water flux, hydrophilicity, porosity, morphological structure, composition, and performance. PTGM and PES/PTGM membranes were characterized by field emission scanning electron microscopy (FESEM), Fourier-transform infrared spectroscopy (FTIR), and contact angle (CA). The results revealed that the porosity and hydrophilicity of the fabricated membrane formed using a 5 wt.% PTGM ratio exhibited an enhancement of 20% and 18%, respectively. Similarly, upon raising the PTGM ratio in the casting solution, a more porous with longer finger-like structure was observed. However, at optimum PTGM content (i.e., 5%), apparent enhancements in the water flux, bovine serum albumin (BSA), and sodium alginate (SA) retention were noticed by values of 203 L/m^2^.h (LMH), 94, and 96%, respectively. These results illustrated that the observed separation and permeation trend of the PES/PTGM membrane may be a suitable option for applications of wastewater treatment. The experimental results suggest the promising potential of PTGM as a pore former on the membrane properties and performance.

## 1. Introduction

Due to the increase in our planet’s population and industrial activities, much fresh water is consumed continuously, which leads to its depletion. Many recent studies have shown great interest in clean water sources in the past few decades. To avoid the freshwater shortage, the scientific community has resorted to focusing on wastewater treatment, which is polluted, to make it usable for various applications such as irrigation and industrial purposes. Many techniques have been suggested to eliminate the contaminants from wastewater at the lowest possible cost, thus relieving the shortage of potential clean water sources. Among the treatment technologies, membrane processes have been presented as an exceptional solution to eliminate contaminants [1]. Moreover, membrane processes can achieve the effective purification of oily wastewater via nanocomposite membranes [2,3].

Membrane technologies have been studied in the past few decades with the latest improvements in the manufacturing method and its impact on the membrane structure. Porous polymeric membranes have attracted much attention for their use in various applications [4].

Ultrafiltration (UF) is a membrane module with a unique separation ability in many industrial applications, including wastewater treatments. In addition, it has low energy consumption, high permeability, and high retention of different contaminants [5]. Fouling is one of the hardest problems that the UF membrane can suffer, resulting in lower performance. Therefore, it became essential to modify the characteristics of the membrane surface to reduce the fouling consequences.

The hydrophilicity and surface morphology characteristics of the membrane were the most impact factors affecting the membrane fouling phenomenon. For better performance, several currently common ways have been used, such as adding nanoparticles in the casting solution [6,7], such as titanium oxide (TiO_2_) [8], zinc oxide (ZnO) [9,10], silica (SiO_2_) [11,12], alumina (Al_2_O_3_) [13], graphene oxide (GO) [14,15,16], and carbon nanotubes [17]; polymeric blends such as polyphenylsulfone (PPSU)/polyethersulfone (PES) polymer blends [18,19], polyphenylsulfone and polysulfone polymer blends [20], and polyphenylsulfone (PPSU)/polyethylene glycol hexadecyl ether (Brij-58) blend membranes [21]; and polymeric grafting such as polyvinyl chloride-graft-poly ethyl acrylate (DHPVC-g-PEA) membranes [22]. Furthermore, water-soluble polymers incorporated as pore-forming additives were another efficient method to modify the membrane characteristics, and among the common water-soluble polymers were polyvinylpyrrolidone (PVP) and polyethylene glycol (PEG) [23,24].

In recent studies, a water-soluble polymer was developed as a novel co-polymer in polymeric membranes to improve the characteristic of the membrane [25,26]. On the other hand, researchers used a novel trend for modifying the membrane to improve its performance and mechanical properties through the use of dual pore-forming additives [27,28,29]. Due to the importance of water-soluble polymer additives in increasing pore density and consequently narrowing the pore size and increasing the porosity, this study will focus on modifying the polymeric membrane using a novel type of water-soluble co-polymer nanoparticles as an additive. Here, novel Poly(Terephthalic acid-co-Glycerol-g-Maleic anhydride) nanoparticles with hydrophilic characteristics have been synthesized and used as a pore-forming agent in a membrane modification. These nanomaterials were harnessed for PES membranes at different ratios to improve them and thus achieve the optimum performance of permeability and selectivity.

BSA and SA were used as solutes to study their rejection behavior. BSA is one of the potential proteins (model fouling agents) that had been given great attention concerning its role in pharmaceutical and biochemistry research. The physical and chemical properties of BSA, particularly its high molecular weight, made it possible to employ the UF membrane process to separate it. SA as an organic foulant model has also been harnessed. It dissolves in water, making it rather hard to separate from the polluted water, which renders the process of its removal difficult by conventional methods.

## 2. Materials and Methods

### 2.1. Materials

Polyethersulfone polymer (PES, MW = 30,000 g/mol) bought from Sigma-Aldrich (St. Louis, MO, USA) was used as the host material of the membrane with a density of 1.37 g/cm^3^. Terephthalic acid and Glycerol bought from Sigma-Aldrich, Steinheim, Germany, were selected as the raw material for preparing the co-polymer. Maleic anhydride was bought from Sigma-Aldrich, Steinheim, Germany, as the raw material for preparing graft polymer. *p*-Xylene was utilized as a remover for the water formed in the esterification reaction process and was bought from Sigma-Aldrich, Steinheim, Germany. Dimethyl sulfoxide (DMSO) and N,N-Dimethylacetamide (DMAC) from the Sigma-Aldrich company (Steinheim, Germany) were utilized as a solvent for polymer dissociation for graft co-polymer and membrane fabrication, respectively. Bovine serum albumin (BSA) and sodium alginate (SA) were brought from Sigma-Aldrich, Steinheim, Germany Germany and chosen as a foulant model. All the chemicals utilized were analytical grade without additional purification.

### 2.2. Experimental Procedure

#### 2.2.1. Fabrication of PolyTerephthalic acid-co-Glycerol-g-Maleic Anhydride (PTGM) Nanoparticle

**Scheme 1:**
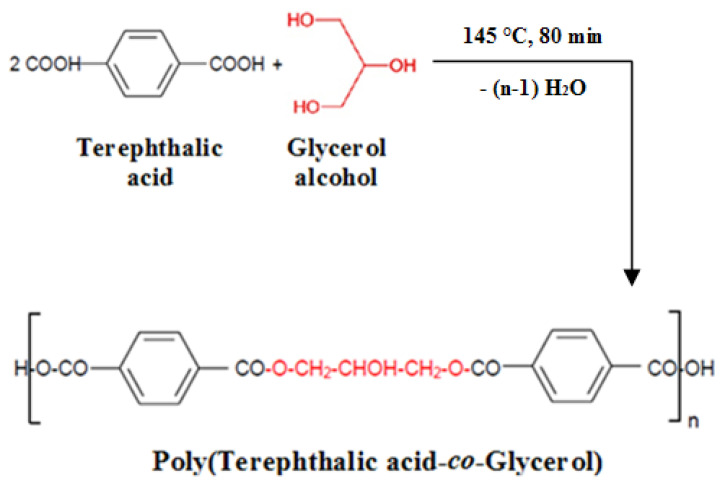
Schematic diagram of the co-polymer preparation.

PTGM nanoparticles were synthesized in two steps. In the first step, 2 moles (332 g) of terephthalic acid and 50 mL of dimethyl sulfoxide were mixed in a stoppered glass conical flask fitted with a thermometer and placed on a hot plate magnetic stirrer. The mixture was heated carefully to 40 °C. After a clear liquor mixture was formed, 1 mole (92 g) of glycerol was added to the solution and heated cautiously to 120 °C. To remove the water formed in the esterification reaction, during the preparation of the nano co-polymer, 25 mL of *p*-xylene was added in the shape of equal batches, 2 drops per batch, to the reaction flask. After 80 min, the heating was stopped without further water coming off (it was at 145 °C). At the end of the reaction, the reaction flask was cooled to 50 °C. This reaction was expressed by Scheme 1.

**Scheme 2:**
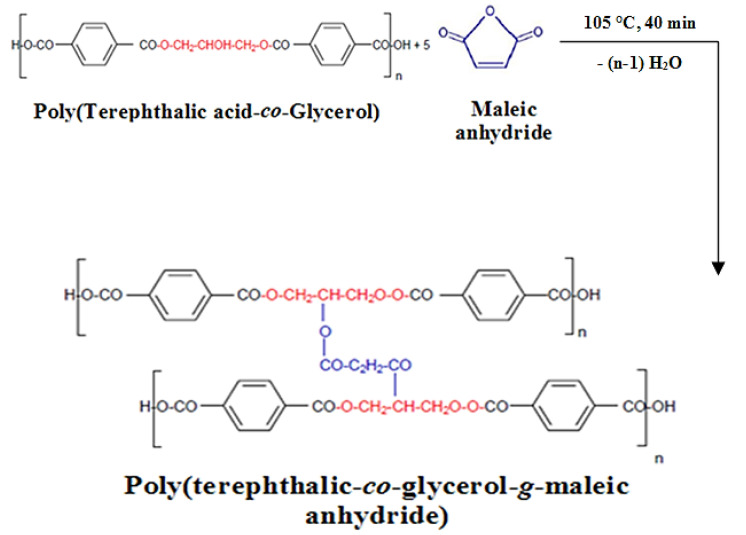
Schematic diagram of the graft co-polymer preparation.

In the second step, maleic anhydride with 0.5 moles (58 g) was dissolved in 10 mL of dimethyl sulfoxide at 40 °C using a hot plate magnetic stirrer. After all the solid was dissolved, the mixture was then added to the previously prepared mixture and gently heated to 90 °C. The intermittent addition of suitable amounts of *p*-xylene to the reaction flask ensures that the water droplets remaining in the solution are removed. By heating at 105 °C for 40 min, the mixture becomes a densely grafted co-polymer known as nano poly(terephthalic-co-glycerol-g-maleic anhydride) solution (Scheme 2). At the end of the reaction, the reaction flask was allowed to cool to ambient temperature and then cold distilled water was added, in which the suspension solution formed after 6 h. The suspension solution was left to precipitate for 24 h, filtrated and washed in pure water, and then left to dry. More details about the preparation method were presented elsewhere [26].

It is important to mention here that this process (partial crosslinking) may completely hinder the formation of hydrogen bonds with water, which leads to the dissolution of the nanopolymer in the form of a hydrogel at a temperature of 20 °C, but when the water temperature is raised to 25 °C, it leads to a complete dissolution of the nanopolymer in the form of a hydrogel in water. Therefore, nano poly(terephthalic-co-glycerol-g-maleic anhydride) is willing to completely dissolve in water.

#### 2.2.2. PTGM Characterization

Proton nuclear magnetic resonance (i.e., ^1^H-NMR) is the application of nuclear magnetic resonance spectroscopy with respect to hydrogen-1 nuclei inside the molecules of a material to define its molecular structure. ^1^H-NMR spectral measurements were acquired on a Bruker DPX 300 device (Billerica, MA, USA) at 300.13 MHz for hydrogen nuclei in CDCl_3_, and all chemical shifts are presented in ppm.

The PTGM graft co-polymer surface topography was examined using AFM (TT-2 AFM) with a tapping mode in the air with an appropriate silicon tip, whereas the data were analyzed with specific software. Moreover, the AFM was used to determine the distribution of the PTGM graft co-polymer particle sizes. AFM was also employed to characterize the surface topography 3D image, roughness, and mean pore size of the PTGM.

#### 2.2.3. Fabrication of Membrane

Seven flat sheets of PES/PTGM nano-polymer membrane were prepared using the phase inversion process. The composition of the membranes was introduced in Table 1. Briefly, 20 wt.% of PES and different amounts of PTGM graft co-polymer were dissolved in DMAC and mixed (at 50 °C during 48 h) until a homogeneous solution was obtained. A proper amount of polymer casting solution was degassed and cast onto a glass sheet with an automatic casting knife. After that, the prepared membrane was moved into a deionized water coagulation container. The formed membranes were washed many times with pure water [24].

### 2.3. Characterization of the Membranes

#### 2.3.1. Membrane Morphology

The structural morphology of the prepared membranes was investigated utilizing a field emission scanning electron microscope (FE-SEM) (SEM, JEOL Ltd. JSM-7100F, Tokyo, Japan) to monitor the morphology of the cross-section and upper surface of the fabricated membranes. The membranes were first cracked in liquid nitrogen and then sputtered with a slim coat of platinum before the cross-section photography with the microscope at 5 kV.

#### 2.3.2. Contact Angle Measurement

A static contact angle (CA) instrument (CAM110, Tainan, Taiwan) was used for hydrophilicity measurements to define the CA by the sessile drop approach. The details were presented elsewhere [28]. For each sample, at least three different locations on the membrane were examined. The average value of these locations for each sample was considered.

#### 2.3.3. Fourier-Transform Infrared Spectroscopy

Fourier-transform infrared spectroscopy (FTIR) (8400 S, Br., Ettlingen, Germany) was used to detect the functional groups of the PTGM nanoparticles and their chemical bonds with the PES membrane. The final spectrum was measured by placing the sample on the machine plate. The measurements were performed with transmittance spectra from 600 to 4000 cm^−1^.

#### 2.3.4. Membrane Porosity Pore Size and Pore Density

The membrane porosity *ε*(%) was estimated using the gravimetric method as stated in Equation (1) [15]. Membrane samples were cut off into suitable pieces, 2 cm by 2 cm, and immersed in distilled water for a day. Following that, wet membranes were taken from the water and dried using blotting paper to remove visible droplets from the surface of the membrane and then weighed to obtain the weight of the wet membranes. After that, the membranes were dried using a vacuum oven for 12 h at 40 °C and weighed to obtain the weight of the dry membranes.

(1) $$\varepsilon \left(\%\right)=\frac{m_{w}-m_{d}}{\left(\rho_{W}\times A_{S}\times \delta \right)}\times 100$$

where *m_w_* and *m_d_* are the wet and dry weight values of the membrane (g), respectively. *ρ_W_* is the density of water at 25 °C (0.998 g/cm^3^), *A_S_* is the effective surface area of the membrane (cm^2^), and *δ* is the membrane thickness (cm).

The mean pore size (*r_m_*) was obtained based on membrane porosity and pure water flux by the Guerout–Elford–Ferry Equation (2) below [12]:

(2) $$r_{m}=\sqrt{\frac{\left(2.9-1.75\varepsilon \right)8\eta \cdot \delta \cdot Q_{W}}{\varepsilon \cdot A_{S}\cdot \Delta P}}$$

where: *ε*, *η*, *δ*, *Q_W_*, *A_S_*, and Δ*P* represent the porosity of the membrane (Equation (1)), the viscosity of pure water (0.00089 Pa.s), the thickness of the membrane (m), the volumetric flow rate of permeate water (m^3^/s), the effective surface area of the membrane (m^2^), and the operational pressure (0.4 MPa), respectively.

Moreover, image J analysis software program (ImageJ ver. 1.50e, US National Institutes of Health, Bethesda, MD, USA) was used in order to estimate the values of the pore density of PES and PES/PTGM membranes.

#### 2.3.5. Membrane Performance

A cross-flow laboratory system was utilized for flux and rejection experiments to study the membrane performance at ambient temperature. The fabricated membranes were cut into a suitable size, fixed inside the module, and compacted using DI water at 4.5 bars for 30 min. The pressure was then reduced to the operating pressure of 4 bars. Membrane permeation was analyzed with a pure water flux (PWF), a 1000 ppm BSA solution, and a 50 ppm SA solution. The permeate flux (*J*) was calculated using Equation (3) below:

(3) $$J=\frac{V}{A_{S}\times \Delta t}$$

where *V* is the volume of the collected permeate (m^3^), *A_S_* is the effective surface area of the membrane in the filtration cell (m^2^), and Δ*t* is the time taken to obtain the volume of the collected permeate (h).

The contaminant’s rejection *R*(%) was evaluated using Equation (4) [12].

(4) $$R(\%)=\left(1-\frac{C_{P}}{C_{F}}\right)\times 100$$

where *C_F_* and *C_P_* were the concentration of contaminants in the feed and the permeate solutions, respectively.

## 3. Results and Discussion

### 3.1. PTGM Characterization

**Figure 1:**
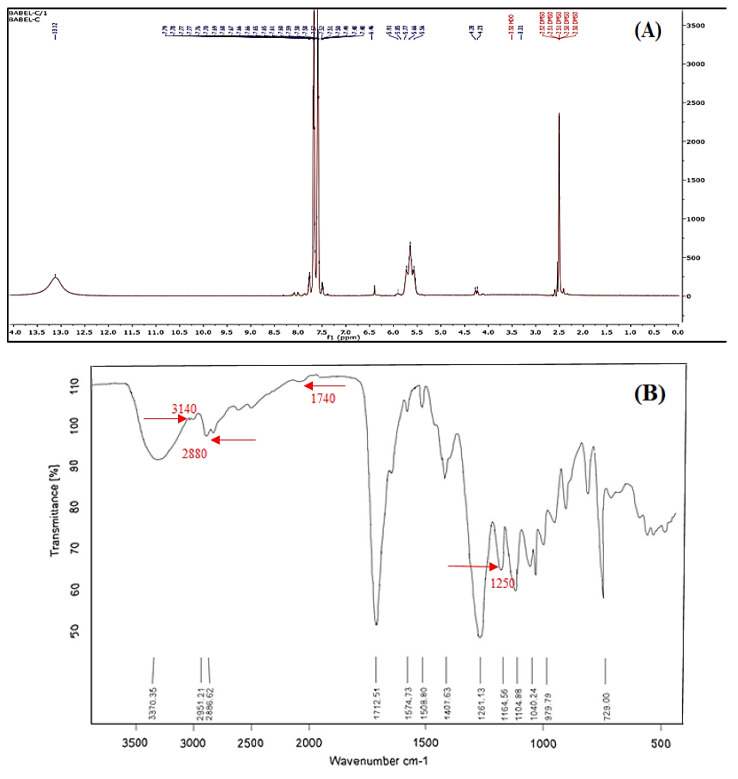
(**A**) The ^1^HNMR spectrum of PTGM graft co-polymer (f1(ppm): concentration of proton in each peak). (**B**) The FT-IR spectrum of PTGM graft co-polymer.

Figure 1 shows the spectrum of ^1^HNMR and the FT–IR spectrum of the graft co-polymers (PTGM). In the spectrum of ^1^HNMR (600 MHz, (CD3)3SO), as shown in Figure 1A, it is clearly observed that the singlet signal at 13.12 ppm referred to the characteristic of (s, protons of carboxylic groups), whereas the signal multiples in the range of 7.79–7.48 was referred to (m, protons of aromatic ring). Moreover, the singlet signal at 6.46 ppm was ascribed to (s, protons of methine group attached to the double bond), while 5.91–5.56 ppm referred to (q, protons of methine groups that attached to oxygen’s atoms), and at 4.28–4.23 it was ascribed to (d, protons methylene groups).

In Figure 1B, it can be noticed from the FT–IR spectrum of the graft co-polymer the formation of the strong broad peak at 3500 cm^−1^ for stretching alcoholic -OH with stretching (H–bond), and the spectrum also revealed the aromatic =C-H, aliphatic C-H, and alkenes =C–H at approximately at 3140 cm^−1^, 2880 cm^−1^, and 3050 cm^−1^ respectively. The spectrum also revealed a strong and sharp peak at 1250 cm^−1^ and 1740 cm^−1^ for C-O ester and a stretching band C=O ester, respectively.

**Figure 2:**
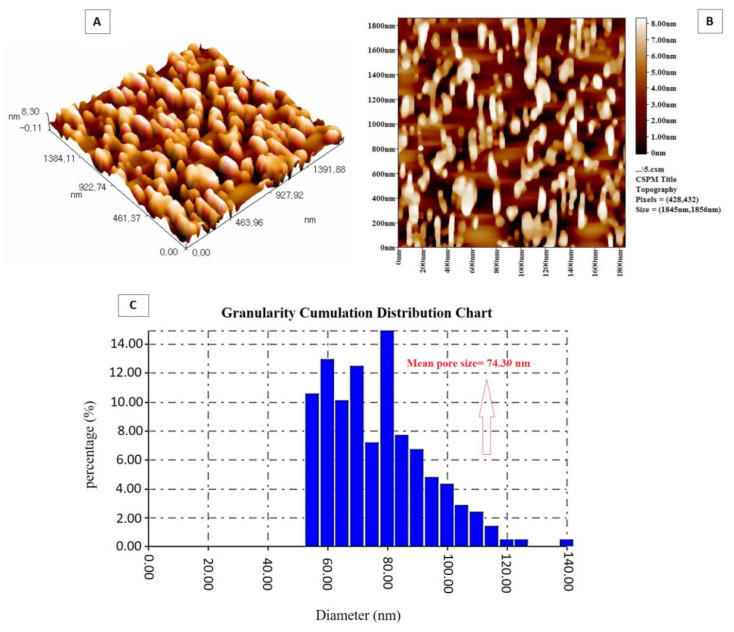
(**A**) Three−dimensional (3D) AFM image of PTGM graft co-polymer. (**B**) Two−dimensional (2D) AFM image of PTGM graft co-polymer. (**C**) Mean particle size and particle size distribution of PTGM graft co-polymer.

The two- and three-dimentional images as well as the mean particle size and particle size distribution of PTGM were estimated by the atomic force microscope (AFM) as depicted in Figure 2. It seems that the PTGM was a graft co-polymer nanoparticle and the mean particle size was 74.39 nm with particle size distribution between 55 to 110 nm.

### 3.2. Impact of PTGM on PES Membrane

**Figure 3:**
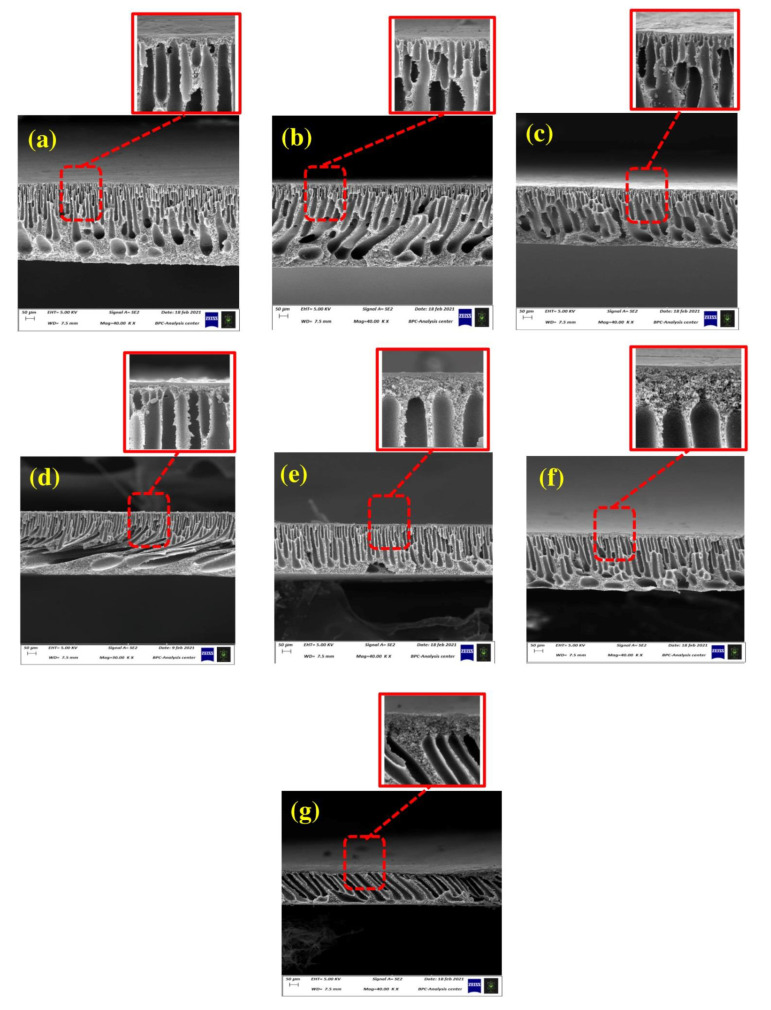
FE-SEM cross-sectional images of (**a**) PES, (**b**) PES/PTGM (1 wt.%), (**c**) PES/PTGM (3 wt.%), (**d**) PES/PTGM (5 wt.%), (**e**) PES/PTGM (7 wt.%), (**f**) PES/PTGM (9 wt.%), and (**g**) PES/PTGM (11 wt.%) membranes.

A critical determinant of performance in preparing the membrane is the incorporation of nanoparticles into the casting mixture to modify the membrane’s structure and, in turn, the membrane’s performance. FESEM cross-sectional images of the prepared membranes by various amounts of PTGM are presented in Figure 3. The finger-like layer took place as a predominant layer of the cross-section. This was because of the comparatively high viscosity of the dope mix caused by the high concentration of polymer used. Noticeably, all the membranes show a characteristic morphology of asymmetric membrane, consisting of a dense top-layer and finger-like layer as well as a thin sponge-like layer near the bottom surface.

The addition of PTGM significantly affects the phase inversion process. The presence of PTGM graft co-polymer increases the solution thermodynamic instability in the coagulation solution (nonsolvent), which facilitates a rapid demixing between DMAC in the casting solution and H_2_O in the coagulation bath and increases the formation of macrovoids. From the other side, it is well known that the complex between PES and graft co-polymer with opposite ionic charges may easily be formed, which decreases the interaction between the chains of the polymer. These phenomena result in an instantaneous demixing, which reduces the thickness growth of the top-layer; therefore, improvement in the macrovoids formation in the sub-layer can be observed. On the other hand, increasing the concentration of the additive (PTGM) will lead to the formation of a highly entangled solution, which leads to an increase in the thickness of the skin layer and the compressibility of the membrane.

The membrane prepared from neat PES (20 wt.%) had a finger-like layer near the top surface, with macrovoids distributed randomly near the bottom surface. Noticeably, the macrovoid walls were relatively thin with closed ends as observed in Figure 3a. The addition of 1 and 3 wt.% of graft PTGM into the casting solution partially modified the morphology of the membrane structure through the formation of a slight and trivial spongy layer at the bottom. Additionally, a semi-regularly distributed finger-like structure with slightly wider pore walls was also observed (Figure 3b,c). An additional amount of PTGM (5 wt.% in the casting mixture) led to increasing the solubility parameter between the solvent and polymer blend, resulting in the elimination of macrovoids and thus the openness of the porous structure. The regular finger-like structure was noticed aligned from the top to the thin sponge-layer observed near the bottom of the membrane (Figure 3d).

The increase in the PTGM content to 7 and 9 wt.% led to the increase in the hydrophilic functional groups in the casting solution, resulting in the acceleration of the diffusion rate between the water and the solvent. Therefore, a wider and longer finger-like structure was obtained with a thick skin layer compared with that prepared from the pristine and low content of PTGM (Figure 3e,f).

A higher additive amount (i.e., 11 wt.% graft PTGM) generated higher hydrophilic functional groups in the casting solution, which, in turn, resulted in the higher penetration of water inside the casting solution throughout the solidification process in the coagulation bath, thus a longer and wider finger-like structure for the whole membrane cross-section was observed compared with all other contents of PTGM in Figure 3g. The effect of hydrophilic groups because of the addition of PTGM was to overcome the effect of casting solution viscosity on the speed of water-DMAc demixing process.

The morphological changes observed as the graft co-polymer (PTGM) increases are logically accepted because a higher PTGM content may result in a higher viscosity of the dope solution. The higher viscosity of the casting solution can induce a lower mixing-demixing between the DMAC solvent and nonsolvent (water) during the phase inversion process.

**Figure 4:**
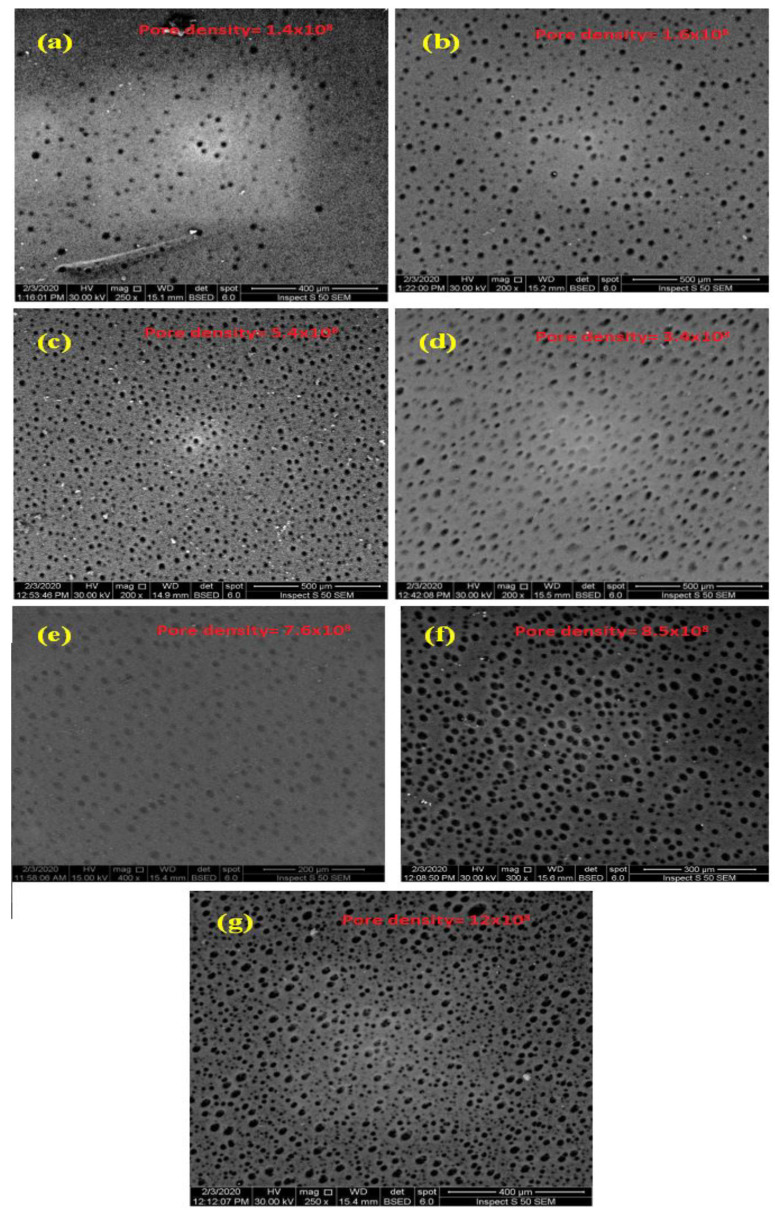
Top surface structures SEM images of (**a**) PES, (**b**) PES/PTGM (1 wt.%), (**c**) PES/PTGM (3 wt.%), (**d**) PES/PTGM (5 wt.%), (**e**) PES/PTGM (7 wt.%), (**f**) PES/PTGM (9 wt.%), and (**g**) PES/PTGM (11 wt.%) membranes.

The top surface SEM images of the PES membranes embedded with PTGM nanoparticles are shown in Figure 4. The onset of pore formation on the skin layer’s surface can be observed after mixing minimal amounts of PTGM nano-co-polymer in the PES solution (Figure 4b). An increase in the pore density and pore size was observed as the concentration of PTGM nanoparticles increased in the polymeric solution (Figure 4c). By continuing to increase PTGM into the casting solution (at 5 wt%), an increase in the membrane porosity and pore size was observed (Figure 4d). The pore density and pore size of the membranes were increased when the PTGM concentration was 7 wt% as depicted in Figure 4e. Further increasing in PTGM content resulted in a significant increase in pore density with wider pore size distribution as depicted in Figure 4f,g. This phenomenon is attributed to the increase in the hydrophilic groups of the casting solutions because of the effect of PTGM, which leads to an increase in the water-DMAc demixing process and thus increases the pore density with wider pore size. On the other hand, the performance and selectivity of the membrane are controlled by the addition of PTGM in the casting solution and the accumulation of PTGM at the interface between the cast membrane and the water during the coagulation phase.

### 3.3. FTIR of PES/PTGM Membrane

**Figure 5:**
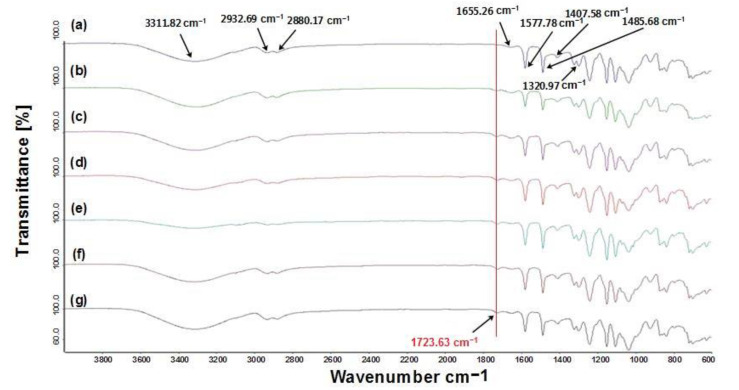
FTIR study of spectra of (**a**) PES, (**b**) PES/PTGM (1 wt.%), (**c**) PES/PTGM (3 wt.%), (**d**) PES/PTGM (5 wt.%), (**e**) PES/PTGM (7 wt.%), (**f**) PES/PTGM (9 wt.%), and (**g**) PES/PTGM (11 wt.%) membranes.

To confirm the existence of the PTGM graft co-polymer in the PES membranes, the Fourier transform infrared-attenuated total reflectance (FTIR-ATI) spectra of the PES membranes before and after the incorporation of the PTGM are presented in Figure 5. Sulfone and benzene groups can be observed on the pristine PES membrane (Figure 5a). Sulfone groups’ vibrations are observed at 1149.93 and 1297.42 cm^−1^; however, the backbone vibrations at 1485.68 and 1577.78 cm^−1^ can be ascribed to benzene groups. The peak at 922.66 cm^−1^ is the peak of aromatic ether (-C-O-C-) chemical bonds.

The characteristic bands of the PES membrane, including a strong, broad peak ascribed to the O–H stretching vibration (3311.82 cm^−1^), medium stretching vibration of C–H band (2932.69 and 2880.17 cm^−1^), C=O alkene stretching vibration (1655.26 cm^−1^), C=C stretching of ester (1577.78 and 1485.68 cm^−1^), O=S=O stretching (1149.93, 1297.42 cm^−1^), and -C-O-C- stretching of ether (922.66 cm^−1^), are observed. Investigating the FTIR spectrum of PES membrane after the embedding of PTGM (Figure 5b–g) does not present any new peaks compared to the pristine PES membrane. This is ascribed to the overlaps of surfactant absorption bands with some of the polymers, except for the peak at 1723.63 cm^−1^, which may be because of the presence of a C=O ester stretch.

### 3.4. Membrane Surface Hydrophilicity, Porosity, and Pore Size

**Figure 6:**
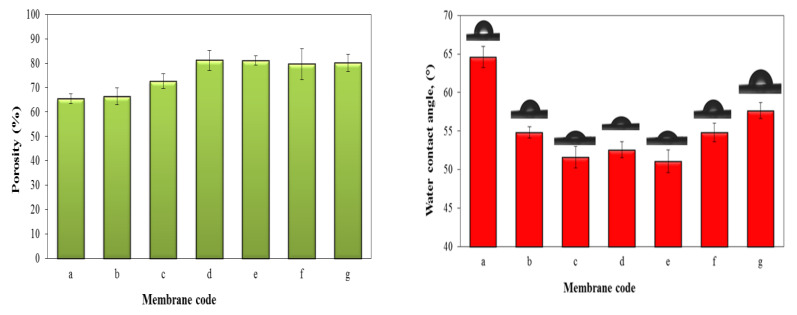
(**Left**) porosity; and (**Right**) contact angle of (**a**) PES, (**b**) PES/PTGM (1 wt.%), (**c**) PES/PTGM (3 wt.%), (**d**) PES/PTGM (5 wt.%), (**e**) PES/PTGM (7 wt.%), (**f**) PES/PTGM (9 wt.%), and (**g**) PES/PTGM (11 wt.%) membranes.

The measurement of the contact angle between the distilled water droplets and the membrane surface gives the surface hydrophilicity. Thus, it plays an essential role in the performance of the membrane [30], whereby a low contact angle indicates a high wettability of the membrane. Figure 6 Left represents the effect of PTGM graft co-polymer content in the polymer casting solution on the porosity of the fabricated membranes. Upon adding 1 wt.% PTGM nanoparticles into the PES polymer dope solution, the porosity of the membrane slightly enhanced from 65.45% to 66.42%. Increasing the PTGM concentration up to 5 wt.% enhanced the membrane porosity up to 81.21%. The modification of membrane porosity following the addition of hydrophilic additives was also reported by Manawi et al. (2017) [31]. They indicated that adding hydrophilic materials in the polymer casting solution constructed an accelerated solvent and nonsolvent exchange rate, which, in turn, showed the modification of a highly porous membrane structure. Adding PTGM showed developing thermodynamic instability in the coagulation bath, hence destroying the dense top skin layer and improving the porosity of the membrane surface. This also progressively altered the pore’s shape from the pore microcavities in the PES membranes to the finger-like pore with a narrower size distribution in the PES/PTGM membranes. So far, the shape of pores in the polymer matrix has been achieved in various ways, including the decomposition of thermally unstable particles from particle co-polymers, the decomposition of thermally unstable particles from polymer blends, or the addition of pore-forming agents (e.g., porogens) during the polymerization process. Higher membrane porosity results in lower resistance to the water flow across the membrane [32]. Higher membrane porosity may be pertinent to a higher pore density. The additional increase in PTGM loading (>5 wt.%) decreased the porosity of the fabricated membrane. This can be analyzed by the increase in the viscosity of the polymer casting solutions, restricting the precipitation process.

Figure 6 (right) shows the contact angles of the pure PES and PES/PTGM membranes. The value of the contact angle of the pristine PES membrane was 64.60°. The contact angle clearly decreased to 54.81° when 1 wt% of PTGM graft co-polymer was added. As the concentration of PTGM in the polymeric solution increased to 3 wt%, the contact angle decreased to 51.59°, which obviously shows an extra increase in the hydrophilicity of the membrane surface. The membrane characteristics showed an improvement in hydrophilicity and wettability when 5 wt% PTGM nano-polymer was added, and the water contact angle value was recorded at about 52.58°. This improvement is affiliated with the increase in pore size and pore size distribution, as well as pore density. The embedding of hydrophilic material could speed up the exchange rate between water (non-solvent) and solvent throughout the solidification process of the membrane, therefore, the porosity of the membrane increases. It is frequently observed that the membrane porosity and hydrophilicity of the membrane has a similar relationship with membrane permeation flux. The development of membrane porosity and hydrophilic character is in favour of the permeation flux improvement. This led to an increment in the nanocomposite membrane hydrophilicity as a result of enhanced porosity. Furthermore, the presence of a higher number of hydrophilic functional groups on the membrane surface that are transported by the hydrophilic PTGM was another main reason for the improvement of the hydrophilic character of the membranes. The value of the contact angle at 7 wt% PTGM is 51.08°. An increase in the amount of PTGM to 9 and 11 wt% revealed a persistent slight increase in the contact angle values to about 54.81° and 57.63°, respectively. This rise in contact angle values correlates with the decrease in porosity obtained at higher dope solution viscosity.

**Figure 7:**
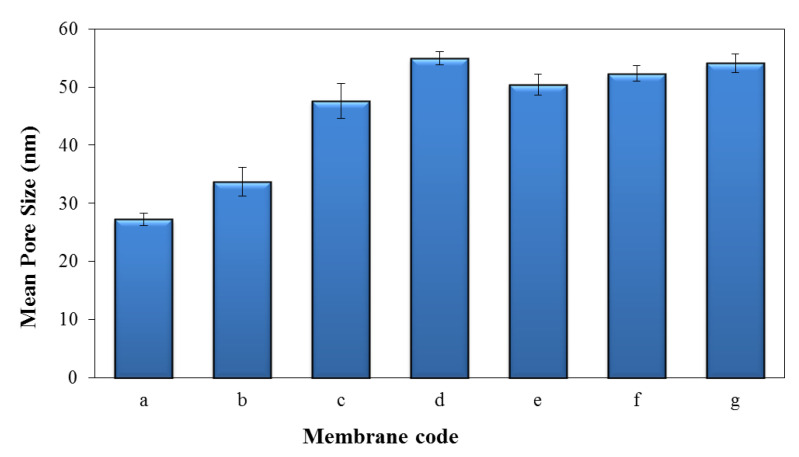
The mean pore size of PES/PTGM membranes.

It is clear that the addition of PTGM nanoparticles to the polymeric solution contributed to the formation of highly porous membranes, as shown in Figure 7. The addition of the previous pore former led to a decrease in the rate of solvent exchange and the formation of pores in large sizes during the separation phase.

### 3.5. Membrane Performance

**Figure 8:**
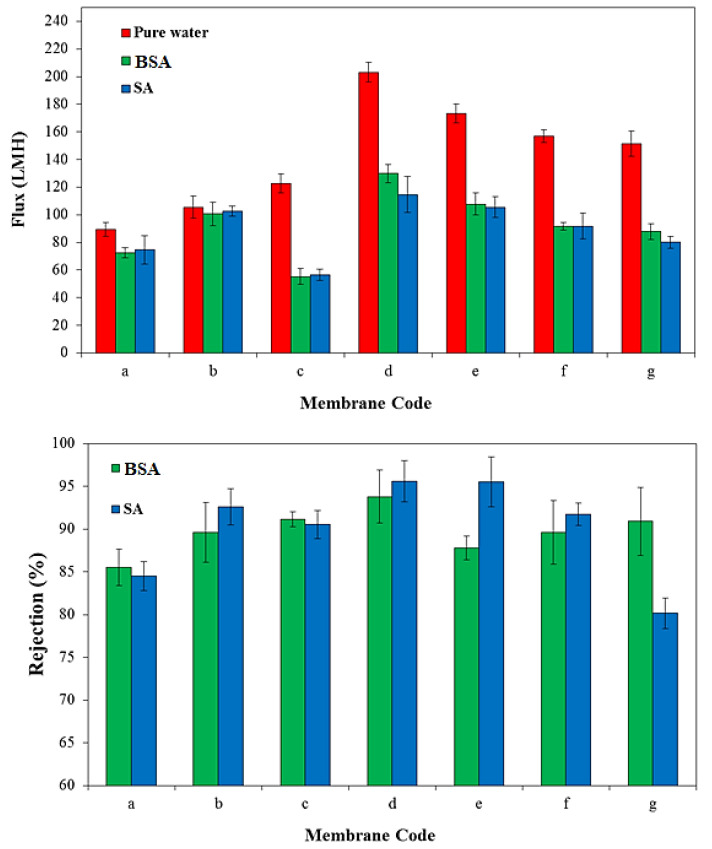
(**Top**) Pure water, BSA and SA flux; and (**Bottom**) BSA and SA rejection of (**a**) PES, (**b**) PES/PTGM (1 wt.%), (**c**) PES/PTGM (3 wt.%), (**d**) PES/PTGM (5 wt.%), (**e**) PES/PTGM (7 wt.%), (**f**) PES/PTGM (9 wt.%), and (**g**) PES/PTGM (11 wt.%).

The top layer porosity and pore density significantly influence pure water flow (PWF) and solute retention [33]. The results confirmed that increasing the content of PTGM nanoparticles in the casting solution leads to a significantly improved PWF of the modified membrane with differences in the separation performance due to changes in the membrane structure (Figure 8). The PWF of the PES/PTGM membranes with different PTGM nanoparticle contents were illustrated in Figure 8 (Top). It was observed that when the PTGM content was increased (0–5 wt%) in the dope casting solution, the pure water flux of the PES/PTGM membranes increased due to surface pore density, porosity, macrovoids, and high surface hydrophilicity, hence the formation of vertically interconnected finger-like pores compared to the rest of the PES membrane [34]. The PWF of the mixed membranes was enhanced as a result of the increase in membrane hydrophilicity, which, in turn, improved the attraction of the water molecules with the membrane morphology [35]. In general, membranes with higher porosity and larger cavities lead to a better PWF of the membrane. The water-soluble polymer nanoparticles accelerate the non-solvent (water)/solvent exchange rate during the phase inversion process, providing a smoother penetration condition for the water molecules. After increasing the PTGM content in the casting solution up to 5 wt%, the highest value of PWF was associated with 20/5 wt% of the PES/PTGM membrane. The results showed that the flow of BSA and SA feed solution across the PES/PTGM membrane decreased sharply compared to the distilled water. The primary reason was that higher-quality PTGM particles resulted in a rougher surface of the fabricated membrane, which was efficiently contaminated by BSA or SA [36]. The retention of the fabricated membranes against the BSA and SA solution is shown in Figure 8 (Bottom). A BSA solution of 1000 ppm and an SA solution of 50 ppm were chosen to evaluate the retention capacity for all experiments. The embedding of PTGM nanoparticles at 5 wt.% exhibited the greatest BSA and SA retention (approx. 95%), compared to 85% for the pristine PES membrane, as can be observed in Figure 8 (Bottom). This practical increase in the BSA and SA removal for the PES/PTGM membranes can be attributed to the influence of the addition of PTGM to the membrane matrix, which imparted a more significant hydrophilic nature to the surface of the membrane. This could have shown lower association and interactions between the membrane surface and contaminants, thus improving membrane rejection. A slight change in this trend was noticed after the addition of PTGM (>5 wt.%) into the dope solution, which led to slightly lowering rejection values. This could be attributed to the decrease in the hydrophilicity of the membrane.

Considering the mean pore size of fabricated membranes from various PTGM (less than 55 nm) and the much smaller molecule diameter of a BSA (less than 7 nm), it is clear that the predominant mechanism for rejecting BSA is not the exclusion of size. In this study, the BSA solution with pH value of 6.7 was higher than the BSA Isoelectric point (IE); thus, the electrostatic repulsive force between the modified surface of the PES membrane and molecules of BSA was increased, which led to a higher rejection of BSA molecules. In addition, the high BSA rejection at high values of pH also may be due to the ellipsoidal shape of the BSA molecules. In fact, the amounts of equilibrium adsorption at the IE point are enhanced around the BSA because of the significant hydrophobic interactions and the lowest electrostatic repulsion interactions between the membrane and BSA molecules and between the BSA molecules itself, thus resulting in the rise in the fouling phenomenon. When the pH value of the BSA solution is above or below the IE point of BSA molecules (IE point between 4.8–5.1), the molecules of BSA became negative or positive charges respectively. Therefore, the BSA charge has slight fouling because of the electrostatic repulsive force with the surface of the membrane depending on the charge of the membrane. It has been observed in the literature that the quantities of BSA adsorption above its IE point are consistently lower than those below its IE point [37].

### 3.6. Membrane Stability

**Figure 9:**
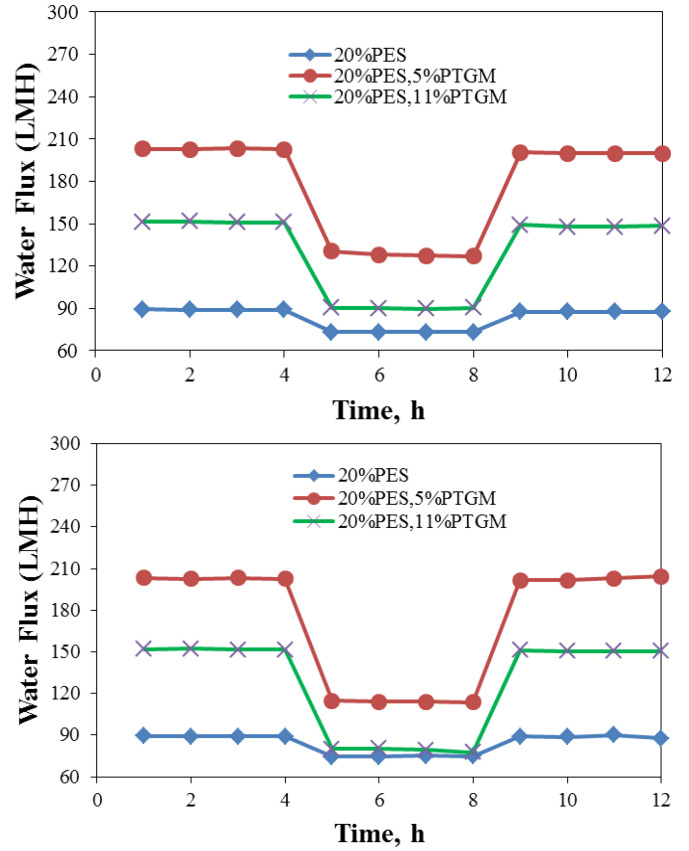
(**Top**) Flux vs. time for the PES/PTGM membranes at 0.4 MPa TMP during the following three steps: water flux for 4 h, 1000 ppm BSA flux for 4 h, and water flux for 4 h after 1 h washing with distilled water; (**Bottom**) Flux vs. time for the PES/PTGM blend membranes at 0.4 MPa TMP during the following three steps: water flux for 4 h, 50 ppm SA flux for 4 h, and water flux for 4 h after 1 h washing with distilled water.

Membrane stability procedures were studied using 1000 ppm BSA and 50 ppm SA solution. Figure 9 shows the time-dependent fluxes of the membranes (PES, PES/PTGM (5 wt.%), and PES/PTGM (11 wt.%)) during three steps of ultrafiltration experiments at 0.4 MPa TMP. The flow of pure water was passed for 4 h and then the feed solution to the membranes for 4 h, followed by the passage of pure water for 4 h after 1 h of washing with distilled water. In general, when pure water was replaced by the feed solution, the permeate flux significantly diminished following the testing of the membranes (PES/PTGM (5 wt.%) and PES/PTGM (11 wt.%)) in all three steps using the BSA and SA feed solution. Furthermore, the pristine membrane (0 wt.% PTGM) showed a low downward trend due to the unmodified nature of the membrane. It can be comprehended that during the filtration procedure, the molecules with a bigger particle diameter in the solution were continuously precipitated on the membrane surface due to the effect of sieving in the surface pores, causing the membrane pores to be clogged. Further, with the continuation of the accumulation, the construction of the filter cake layer on the membrane surface was additionally promoted, which restricted the penetration of the feed solution. Furthermore, the water flux can be fully recovered after membrane cleaning, which meant that the impact of the irreversible pollution can be completely destroyed by cleaning. The flux recovery ratio was estimated to see the extent of contamination appearing on the membranes. The membranes with PTGM additives displayed an excellent flux recovery ratio value, with the highest value being approximately 97% using BSA feed solution and 98% using SA feed solution. However, this shows that modifying the membranes via PTGM improved the good antifouling performance.

### 3.7. Comparison Study

Table 2 illustrates the performance comparison of PES/PTGM membranes prepared in the present work with different pore formers presented in the literature. The important characteristics and performance of membranes are also presented in Table 2. It appears that the efficiency of the novel PES/PTGM membranes has optimum values compared to the water-soluble pore former for membranes that are more widely used in the literature.

## 4. Conclusions

Mixing water-soluble nanomaterials as pore former agents in the polymeric casting solution is another common approach to improve membrane surface characteristics and thus improve its performance. PES ultrafiltration membranes have been optimized using a novel water-soluble nanopolymer (PTGM) for separation applications. This approach aims to restructure the membrane performance using the classical phase inversion technique to prepare different membrane structures. A significant effect on the structure, surface features, and performance of the pristine and PES/PTGM nanoparticles was observed using different percentages of PTGM content (0, 1, 3, 5, 7, 9, and 11% by weight). The obtained results indicated that increasing the PTGM content in the casting solution enhanced the permeability of the composite membranes due to the improvement of porosity, hydrophilicity, and pore density.

The fabricated membrane permeability was considerably improved while having good BSA and SA rejection. When the loading ratio of the PTGM nanoparticles was 5%, the PWF of the modified membrane reached up to 203.07 L/m^2^.h, while rejection was close to 94% and 96% for the BSA and SA solution, respectively. According to the obtained results, employing PTGM as a water-soluble pore former for the PES membrane could construct a novel membrane that can resist harsh operating conditions and can be used for long-term industrial operation in wastewater treatment applications.

The preparation of a novel water-soluble PTGM graft co-polymer can be considered an excellent way to modify the structural morphology of UF membranes in order to overcome the fouling phenomenon of the membrane.

## Figures and Tables

**Table 1:** Composition of the prepared casting solutions of PES/PTGM membranes.

Membrane Code	PES (wt.%)	DMAC (wt.%)	PTGM (wt.%)
a	20	80	0
b	20	79	1
c	20	77	3
d	20	75	5
e	20	73	7
f	20	71	9
g	20	69	11

**Table 2:** Comparison of the results observed by this work with the recent works found in the literature.

Porous Membrane		Contact Angle (°)	Porosity (%)	Mean Pore Size (nm)	Pure Water Flux (L.m^−2^.h^−1^)	Rejection (%)	Process	Ref.
Polymer	Pore Former							
20% PES	5% PTGM	52.58	81.21	54.91	203.1	93.8% BSA 95.6% SA	UF	This Work
22% PVDF-HFP	5% CNS	87	89.9	NA	35	>99.9% MO	UF (MD)	[17]
20% PES	4% TGF	50.4	73.3	40.59	300	96% BSA	UF	[26]
15% PES	2.5 g PMG	42.04	83	108.28	908	98% BSA	UF	[38]
15% PVC	0.119% MWCNT-g-GO	13.9	81.4	259	254	88.9% COD	NF	[16]
19% PES	2% SLS-CNT; 15% PVP	57	74	64.29	597	95.6% BSA	UF	[39]
17% PES	5% PEG 400; 2% Tween-20	NA	35.31	73.2	36.9	93.3% BSA	UF	[40]
PVDF-HFP	4% LiCl; 10 wt.% glycerol	79	NA	7.85	51	90% Aqueous solution	UF	[41]
PVDF	MOF-199/PEG	85	80.89	50	185.05	94% BSA	UF	[42]
20% PES	0.5% CC–Fe_3_O_4_; 1% PVP	52.5	86.3	5.5	36	99% Dye	NF	[43]
0% PES; 14% PAN	4% PVP; 0% DEG	76	55	NA	100	92.47% Humic acid	UF	[44]
15% PSF	0.7% SiO_2_	71.3	78	10.7	55	99.1% NaCl solutions	RO	[45]

## Data Availability

Data is contained within the article.
